# The Commit to Be Fit framework: a community case study of a multi-level, holistic school-based wellness initiative in rural Virginia

**DOI:** 10.3389/fpubh.2023.1067454

**Published:** 2023-08-16

**Authors:** Alisha H. Redelfs, Madeleine Smith, Jacinda A. Merrill, Shannon Grimsley, Hollyann E. Jenkins, Jacqueline S. Tederick, Amanda G. Butler, Kirsten Dueck, Margy Eastham Thomas, David A. Perez, Leah D. Whigham

**Affiliations:** ^1^Department of Public Health, Brigham Young University, Provo, UT, United States; ^2^District Central Office, Rappahannock County Public Schools, Washington, VA, United States; ^3^PATH Foundation, Warrenton, VA, United States; ^4^Sun City Dietitians, El Paso, TX, United States; ^5^Center for Community Health Impact, The University of Texas Health Science Center at Houston (UTHealth), El Paso, TX, United States; ^6^Department of Health Promotion and Behavioral Sciences, The University of Texas Health Science Center at Houston (UTHealth) - School of Public Health, El Paso, TX, United States

**Keywords:** social ecological model (SEM), child health, school intervention programs, holistic health, Whole Child, healthy schools, school wellness, Health Belief Model (HBM)

## Abstract

**Background:**

Public health interventions that target children's physical, mental, and emotional health will enhance their ability to learn and grow. Although more complex, school initiatives that address multiple ecological levels and take a holistic view may be more effective and likely to lead to lasting change.

**Aims:**

This article presents the framework of Commit to Be Fit (C2BF) as an example of how schools can integrate multi-level and holistic approaches for health. This innovative school-based intervention includes activities addressing individual, home, school, and community to create a culture of wellness. We describe the implementation of C2BF and its basis in ecological models and give examples of activities across three components: cafeteria, classroom, and community. We discuss challenges and note that leadership engagement and alignment were critical elements for C2BF's success thus far.

**Discussion:**

C2BF uses a school-based multi-level approach to creating a culture of wellness and holistic health for students, teachers, and community members. C2BF is unique compared to other school-based programming and includes activities that address all eight domains posited for program sustainability within public health. Built to be flexible and adaptive, C2BF was able to successfully pivot during the COVID pandemic and also follow new science.

**Conclusion:**

C2BF and other multi-level holistic approaches are more likely to achieve long-term change by utilizing strategies across the multiple levels of the ecological model to improve health and wellbeing.

## 1. Introduction

The United States is experiencing negative trends in children's health. Children experience worse mental health, decreased resilience, an increasingly sedentary lifestyle, and higher rates of obesity when compared to previous generations (1–3). An inverse relationship exists between child mental health and academic achievement. Results from a 20-year longitudinal study indicate that mental health problems (e.g., internalizing such as anxiety or depression; and externalizing such as conduct disorders) predict educational attainment (e.g., academic performance in English and mathematics and number of incomplete final grade levels from compulsory school) (4). In addition, excess weight gain in children can create a snowball effect of interrelated health issues with emotional, psychological, and social impacts (5). Without effective efforts to counter such trends, many children will continue to experience a substantial health gap (6, 7) with downstream effects such as lower pay and discrimination (8), decreased life span, lower quality of life, and increased rates of depression later in life (9).

Best practices for addressing the complexity of child health include engagement in activities on more than one ecological level (multi-level approach) and addressing more than one aspect of health (holistic approach). In behavioral ecological models, multiple levels of influence interact across levels, and the most effective approaches for changing behavior will address multiple levels (10). Single-level interventions may create short-term effects but tend to be less robust or sustainable in their impact (10). Interventions with multi-level approaches can incorporate participation from peers, teachers, parents, and community members, leading to increased momentum and broad intervention support (10, 11). Multi-level school-based approaches decrease childhood obesity (12) and improve mental health (13, 14), especially when community members are also involved (15). Such approaches tend to cover intrapersonal (in this case, the student), interpersonal (teachers or family), and school levels. On the other hand, interventions using holistic approaches that address multiple aspects of student wellbeing (e.g., physical, mental, and emotional needs and academic achievement) have also been successful (16). The “Whole Child” initiatives that have become popular across the US in recent years are examples of holistic approaches to addressing child health (17–19). To date, we have not found examples of school-based child health programs that combine multi-level strategies with a holistic approach to health for students as well as teachers and community members.

Schools are uniquely positioned to influence childhood health; most American children (50.8 million) spend an average of 7 h per day at school. A “health promoting schools” approach can influence students' activity levels, eating habits, and mental health (20–24), but this requires a coordinated effort and cannot be effectively addressed using oversimplified interventions (21).

This paper describes the Commit to Be Fit (C2BF) framework as a community case study of how a school may combine best practices to address health holistically by integrating activities at the individual, home, school, community, and policy levels to create a culture of wellness for students, teachers, and the community. This paper is not a comprehensive evaluation of C2BF nor an in-depth review of all pertinent literature.

## 2. Context

Rappahannock County Public Schools (RCPS) is a small Virginia school division in a rural county (population 7,500), with one K-7 elementary school (~500 students, ages 5–12, one class of preschool age 4) and one high school (~320 students, 13–18). Rappahannock County has limited access to food and wellness resources; 74.8% of the residents do not have access to a large grocery store (25), and prior to C2BF, many residents would travel by car for upwards of 30 min to reach a full-service gym, wellness center, or medical clinic. Only 1 in 3 adults attended college, and 1 in 3 homes do not have internet access (26); there is a wide gap in income and access to health resources in the county.

### 2.1. History and development

Commit to Be Fit (C2BF) is an award-winning school-based holistic wellness program at RCPS. The program aims to improve the overall (physical, mental, emotional, and social) health and wellness of students, parents, staff, and community members (https://www.rappc2bf.com) by creating a healthier culture. C2BF was piloted and implemented with funding from a regional health foundation. The initial C2BF team included the superintendent (principal investigator), the district's nutrition director, and two wellness integration specialists certified as health coaches, fitness instructors, and action-based learning facilitators. Initially, C2BF was framed as addressing childhood obesity but pivoted to a more holistic approach and is now focused on building a “culture of wellness” within the school division and among the community.

C2BF is unique in multiple ways. First, C2BF uses a three-pronged approach (cafeteria, classroom, and community components) to work simultaneously across multiple facets of health and multiple ecological levels. Addressing wellness at the community level and focusing on students/school staff was especially important due to disparities in access across the county. Second, the superintendent initiated this effort, got district leadership to buy in early, and continues integrating C2BF into general district practices and policies. Finally, C2BF was designed to be implemented progressively over 5 years, addressing different themes and ecological levels each year to build outward over time: (1) Inspiring Healthy Role Models (teachers and school staff); (2) Focusing on the family; (3) Changing Community Culture; (4) A Broader Scope; and (5) Global Outreach and Sustainability. The smaller initial scale allowed C2BF to build stakeholder buy-in gradually and test the synergy of the interconnected elements before scaling up and adding further layers of complexity.

C2BF was the recipient of the 2017 Virginia School Board Association's *Food for Thought Competition* for wellness/physical activity. Over a dozen presentations on C2BF's model have been given at various local, national, and international education, health, and wellness conferences (27–29).

## 3. The Commit to Be Fit model

### 3.1. Guiding theory

**Figure 1:**
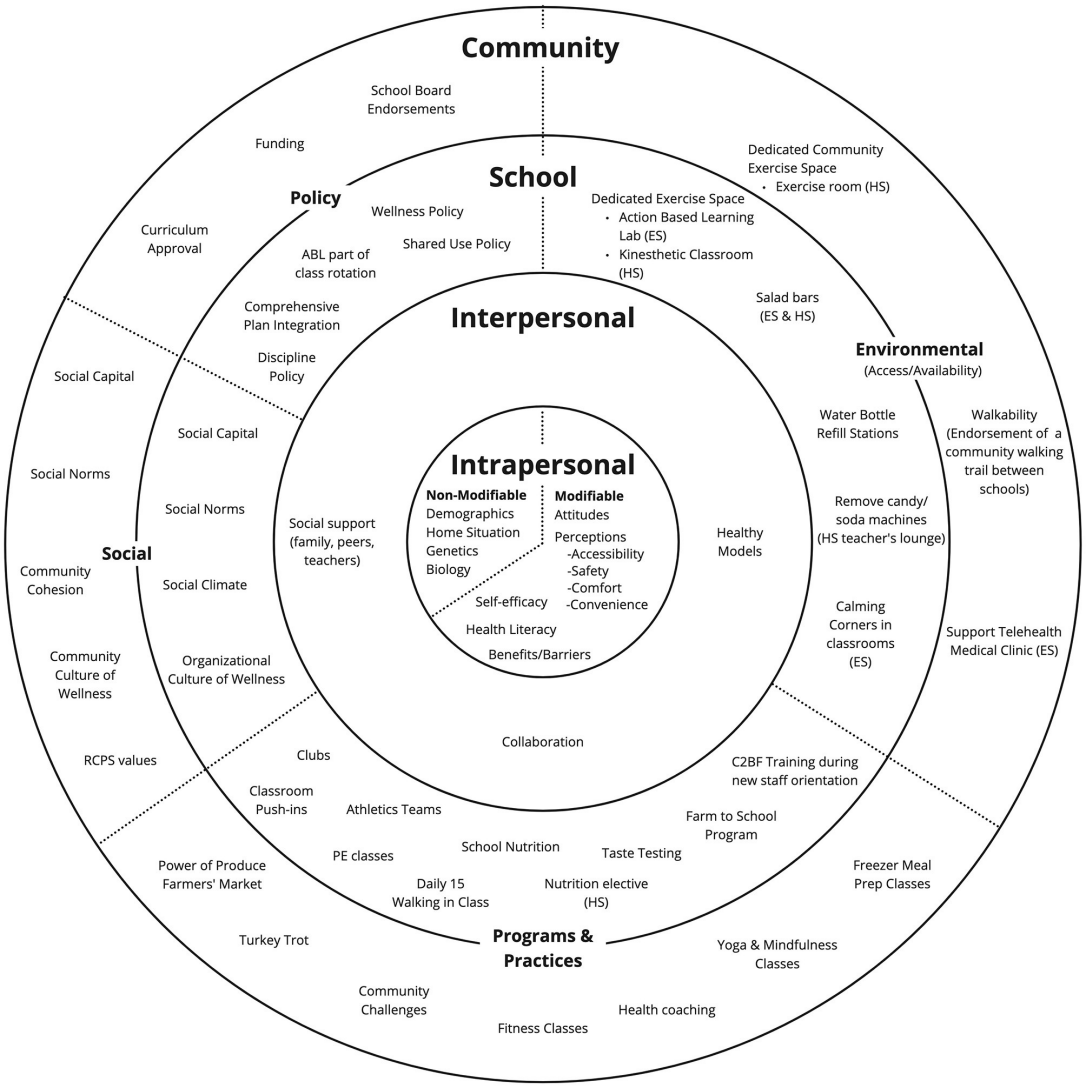
Ecological model for C2BF. ES, Elementary School; HS, High School; PE, physical education.

C2BF is based on the social-ecological model and the Health Belief Model. Because health issues, such as poor mental health or obesity, are complex and are caused by multiple factors, using an ecological framework that encourages intervention at multiple levels is critical (56) to address the underlying factors influencing health (30). C2BF influences the health of children and adults in Rappahannock County by intervening at the intrapersonal (individual), interpersonal, school (organizational), and community levels (Figure 1).

C2BF activities addressing each level are intended to influence intermediate outcomes within a particular dimension (shown in Figure 1 as wedges: social change, policy, environmental change, and programs and practices) that may intersect with and influence other behaviors or audiences. For example, C2BF offers fitness classes and challenges at the schools to build a culture of fitness among teachers, who then may impact social norms at the interpersonal level (e.g., via modeling) to change student perceptions and encourage students to also engage in healthy behaviors (e.g., reducing inactivity).

The Health Belief Model was also considered when developing C2BF programming at the individual level. Constructs such as perceived benefits, perceived barriers, and self-efficacy (31) help explain why people desire and engage in certain health behaviors (11, 32). An example from C2BF is a class on preparing healthy meals to freeze. Learning about the benefits of increasing fruit and vegetable intake, modeling, and practicing can increase self-efficacy in preparing healthy, easy-to-make meals at home. Perceived susceptibility and perceived severity help people to gauge risk; C2BF components regularly apply these constructs by addressing the connections between behaviors and disease as well as between behaviors and health (e.g., health coaching, newsletters, etc.).

The application of behavioral theory can help maximize the likelihood of behavior change. Using a tool developed by a team of health pedagogy and behavioral theory experts to focus curricular efforts on essential knowledge and skills needed to support the adoption and maintenance of healthy behaviors (see https://sph.uth.edu/research/centers/cchi/resources), the *Personal Fitness and Nutrition* course curriculum (high school) was assessed for best practices in Year 2. Lesson plans were developed or modified per the National Health Education Standards, objectives from the Health Education Curriculum Analysis Tool, targeted Healthy Behavior Outcomes established by the Centers for Disease Control and Prevention, and The Characteristics of Effective Health Education (33–35). For example, one lesson plan addressed the connection between nutrition and chronic disease and calories based on grams per macronutrient. Several additional activities were suggested to address functional knowledge, self-efficacy, perceived susceptibility, perceived severity, and skill while drawing the connection between nutrition and disease and aligning with the above health education standards and resources.

- Using fictional case studies, students determine possible nutrients lacking in patients that may have contributed to their disease development.
- Students create an online family health history tree using *My Family Health Portrait: A tool from the Surgeon General* (https://cbiit.github.io/FHH/html/index.html) and summarize (a) chronic diseases that run in their family and (b) nutrition behaviors they can practice themselves to help prevent the development of chronic disease.

### 3.2. Initiative components

**Figure 2:**
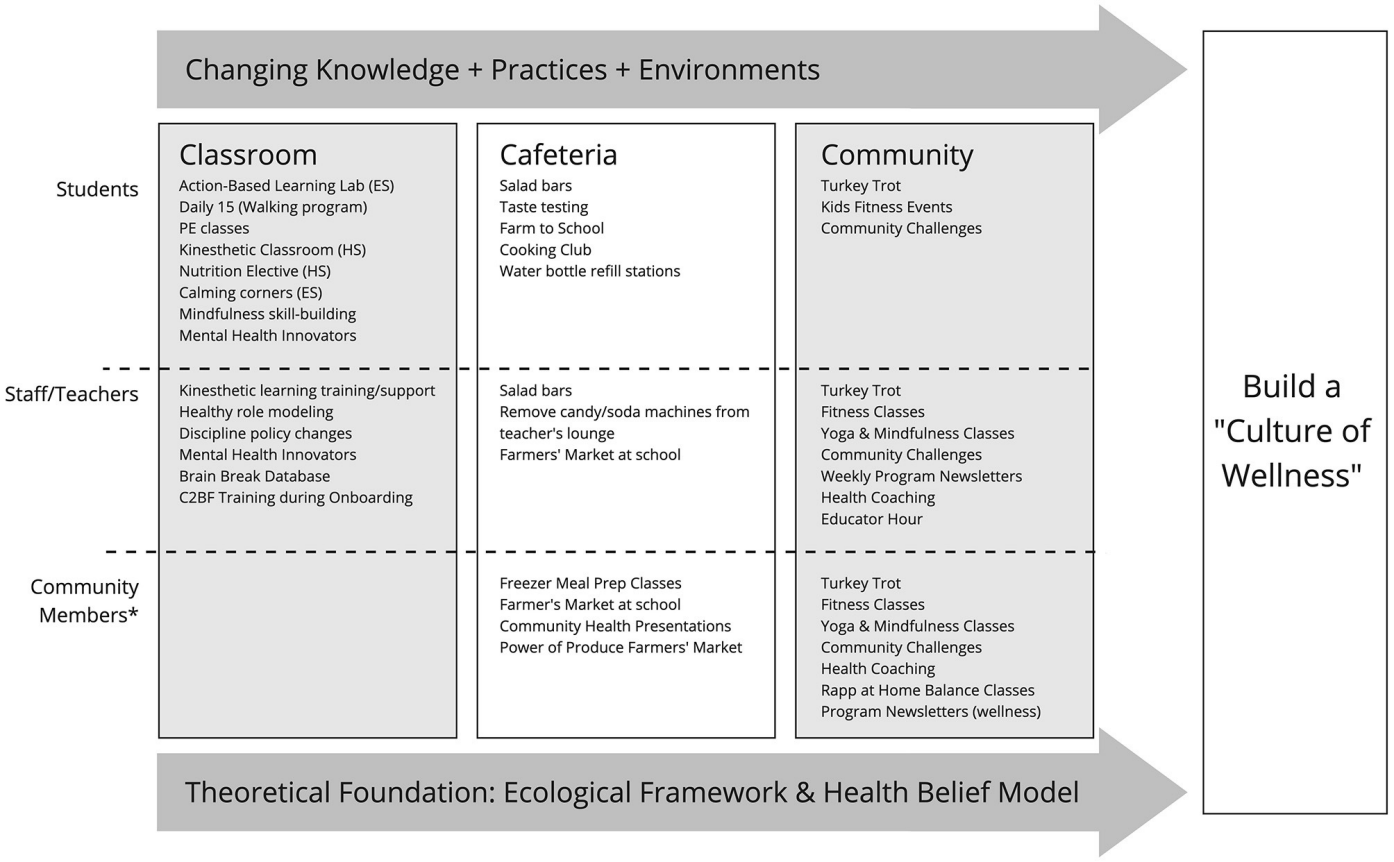
C2BF program activities by audience and component. ES, Elementary School; HS, High School; PE, physical education.

C2BF activities are designed to build a “Culture of Wellness”, so activities are implemented among three audiences (students, staff/teachers, and community members) and across the three components (classroom, cafeteria, and community). Figure 2 is a matrix that lists activities within these components and across the three audiences to illustrate the scope of C2BF. The ultimate intended beneficiaries of C2BF are the children, with children's health most likely to be achieved when there is overall wellness in staff/teachers, parents, and the community.

#### 3.2.1. Cafeteria

The cafeteria component and its associated activities strive to enhance school nutrition to contribute to a “Culture of Wellness”. For example, at the community level, the C2BF team created a farmer's market at the school: twice a year staff members are given vouchers as a show of appreciation from the C2BF team and district administrators. Students are provided “pop bucks” to purchase a complete meal (e.g., protein and produce) from the weekly Power of Produce collaboration with the Farmers Market between April-Oct. Benefits include increased connection among staff and administrators (social climate and collaboration); improved accessibility of fresh produce (e.g., during summer vacation for students and intrapersonal produce intake); introducing students, staff, and parents to the market; as well as providing financial support to local farmers, and building relationships between community members and farmers (community cohesion).

Several additional activities address psychosocial and behavioral factors at the intrapersonal level. Taste testing (monthly) helps students experience a variety of foods and has been shown to change food preferences by addressing food neophobia since children can be scared to try new foods (36). A school garden may help students appreciate food sources or be more willing to try new feeds (37), while an elementary school cooking club can build self-efficacy for preparing healthy foods (38). At the school level, adding daily salad bars and incorporating Farm to School (an environmental change at the school level) increased fresh produce availability for students and teachers (environmental change and increase determined using cafeteria records).

#### 3.2.2. Classroom

At the school level, the classroom component uses kinesthetic learning techniques to improve academic achievement and incorporates additional physical activity opportunities to decrease sedentary time (see Supplementary video). The Action Based Learning (ABL) lab is a converted classroom at the elementary school with a series of 10 stations designed to help fill developmental movement gaps (e.g., activities that encourage crossing the body midline) and enhance learning by improving brain functioning (Action Based Learning by Kids Fit; https://www.abllab.com and https://www.ablacademy.com). ABL was added to the exploratory class rotation (e.g., art and music), so children use the lab for 30 min once every 6 days per school policy. The lab also allows traditional classroom curricula to be taught with kinesthetic methods and is designed for practicing mindfulness. The “Neuronasium” is a converted classroom at the high school, outfitted with special desks that are paired with cardio equipment to allow students to move while learning (e.g., pedals and gliders). Specific classes such as *Personal Fitness and Nutrition* are taught in the Neuronasium each school year, and any teacher can schedule time there for other classes as well. During a 2020 visit from the Assistant Secretary of the US Department of Education to RCPS, Dr. Dave Meyers (Assistant Superintendent for Data, Research, and Technology at the Virginia Department of Education) stated, “I have been through thousands of schools and have never seen anything like the Neuronasium before” (personal communication, September 17, 2020). Additional class activities were incorporated in both elementary and high schools to build skills such as emotional regulation (e.g., calming corners), mindfulness, and resilience in students and teachers at the intrapersonal level. C2BF staff also offer annual trainings for teachers (e.g., brain breaks), and are available to facilitate kinesthetic learning time in individual classrooms upon request (e.g., push-ins where C2BF staff join a class and facilitate the activity, offered daily). Such trainings have not been required; instead, the strategy was first to work with innovators and early adopters and develop champions. Later, they expanded their focus to the early and late majority when those teachers were more ready (39).

#### 3.2.3. Community

Lastly, the community component aims to improve the health and wellness of the staff, parents, and other community members through free fitness classes, events, workshops, and various incentives. For example, trauma-informed yoga and mindfulness classes are provided weekly; according to feedback from community members, participation in these sessions increased confidence in using these tools to improve mental health and build resilience skills. Additionally, converting the high school teachers' lounge into an exercise space allowed C2BF to offer free physical activity classes (e.g., step, balance, cardio boot camp, and circuit) to community members, an environmental change that increased access in this rural county. Weekly “Wear Your Workout Clothes to Work Wednesdays” encourages teachers to engage their classes in a movement activity with their students while simultaneously decreasing a barrier to engaging in physical activity themselves. Engaging with this extended audience (parents, staff, and others in the community) is strategic: (1) to get students on board, you need parents and teachers to be on board and (2) culture is best changed with a community approach (40).

**Figure 3:**
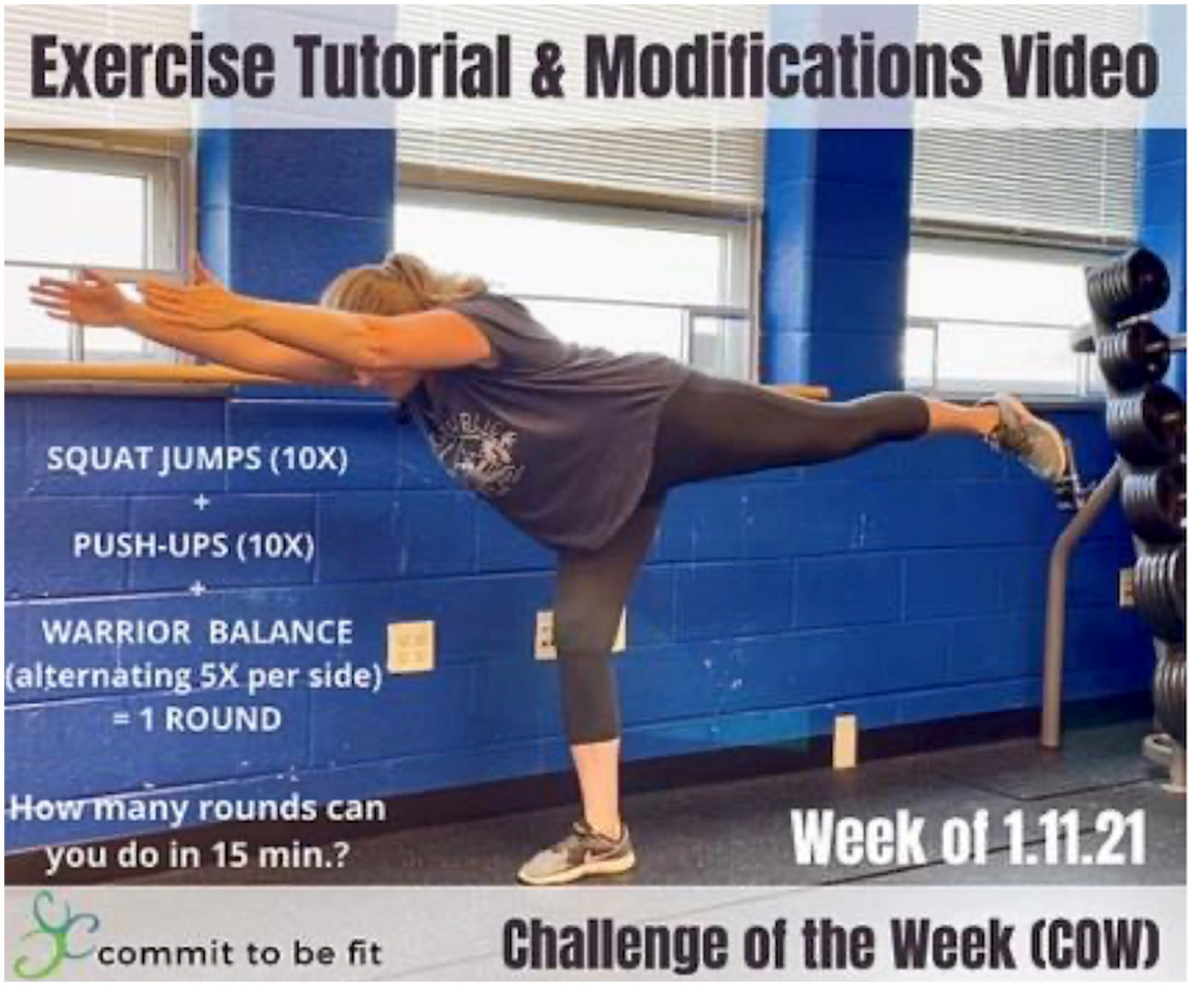
Example of the C2BF challenge of the week (online exercise tutorial and modifications).

Other examples of community activities include monthly community challenges. The first walking challenge, created as a response to COVID pandemic stay-at-home orders, used social media accounts to register teams from the community to “Step on Hunger”, with C2BF committing to donate funds to the local food pantry based on the total distance walked. What started as a goal to “walk to San Francisco” became a series of walking challenges that resulted in enough miles walked to reach Australia over 8 weeks. The online Challenge of the Week has persisted (Figure 3), and the C2BF staff has built a significant repository of free wellness, exercise, and mindfulness videos and worksheets available to the community.

### 3.3. Process: scope, delivery, and reach

The various program activities (Figures 1, 2) in C2BF were intentionally chosen to fit within the revised scope. The team differentiated roles for responsibility and delivery of activities and sessions. Broadly speaking, the Nutrition Director oversaw efforts related to nutrition such as taste testing and Farm to School, the C2BF coordinator supported community efforts such as setting up the partnerships to hold Farmer's Markets at school, and the C2BF communications specialist oversaw program newsletters and social media programming. Because of the multi-component nature of C2BF and the use of communications as part of the interventions within the community, it can be difficult to quantify the full reach of the program in terms of independent persons touched. However, programming occurred for both students and employees across the school division (including both the elementary school and high school) in every year since 2016. Student body size across the two schools averaged 807 students (min 747, max 884 students), and about 164 employees per year (77 elementary; 53 high school; 34 central office, other). Participation in activities can range widely, from under 10 individuals participating in an after-school exercise class to hundreds participating in teams during the 2020 C2BF walking challenge (see Supplementary material C2BF Program Delivery and Reach for a list of activities, responsibilities, frequency, and estimates of reach).

One economic limitation of the current C2BF model is the reliance on external grant funds for team salaries. The C2BF coordinator, the nutritionist, and ABL coordinator were all fully grant funded. The food service director was fully funded by RCPS. However, the superintendent has begun institutionalizing positions related to C2BF; the communications director is covered at 50% by RCPS.

### 3.4. Flexibility and challenges

C2BF was designed with pre-determined themes to expand the program's reach over time. The initial intended long-term goal of C2BF was to reduce childhood obesity. There came a growing understanding that obesity is a multi-faceted disease, with influences including genetics and environmental stressors as well as dietary and lifestyle choices (41), and recognition that obesity is challenging to address. Additionally, initial implementation of C2BF led the team to recognize the interplay between physical, emotional, and mental health that drives overall wellbeing was the true intent of the program. While reducing the risk of childhood obesity might be an appropriate goal for a subset of the students, holistic wellness became the preferred focus of the team.

As the focus evolved, activities were added each year, including efforts to expand partners and achieve community buy-in. Furthermore, because C2BF was intentionally designed to allow for adaptation and modification, innovative ideas that matched the intent and goals of C2BF could be adopted. For example, C2BF created a sensory pathway in the elementary school by adding stickers on the floor spanning the length of two hallways, encouraging students to engage in various movement patterns (e.g., hopping, marching, heel-to-toe balance walk, hopscotch, and crab walking) during breaks and to and from recess. The flexibility that allowed C2BF to pivot toward holistic wellness and incorporate new ideas was also critical to successfully navigating the problems and challenges that arose over time, including during the COVID-19 pandemic.

**Figure 4:**
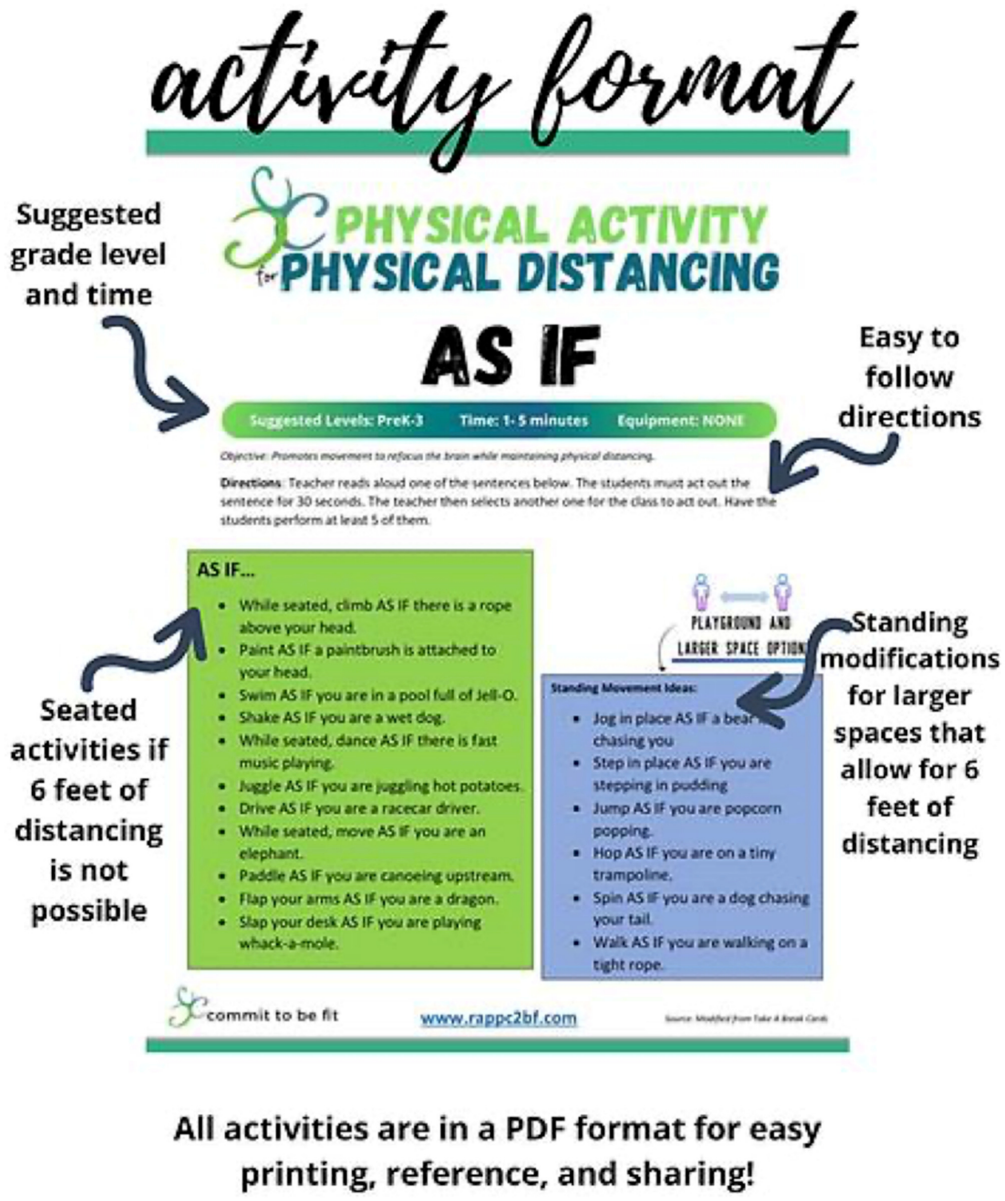
Example of physical activities class activities created by C2BF for teachers as modified for COVID-19.

Implementation very rarely occurs without challenges arising. A small sample of the barriers to implementing C2BF are outlined in Table 1 based on a series of fall 2022 individual interviews with the C2BF staff and RCPS Superintendent. The table also lists how the barrier impacted the program and the actions the C2BF staff took to overcome these challenges. The C2BF team and leadership were creative and strategic in addressing barriers from limited space to resistance to change. The global COVID-19 pandemic required the C2BF team to pivot significantly. However, several modifications—such as the highly successful online walking challenges and modifications for classroom physical activity (Figure 4)—allowed programming to reach a different audience within the county than had been previously connected to C2BF. Additionally, the efforts the C2BF team had made to begin helping students, teachers, and community members develop emotional regulation skills and mindfulness before the pandemic became even more critical. For these reasons, an additional portion of the C2BF staff time was shifted toward activities to improve resilience and mental health during the 2021–2022 and 2022–2023 school years.

**Table 1:** C2BF challenges, impacts, and actions taken.

**Challenge**	**How it impacts C2BF**	**Actions taken by C2BF team**
Dynamics of Rappahannock County (e.g., size of county and distance traveled)	Difficult to reach the entire county and for community members to participate in organized physical activity	• Increasing opportunities available to community members (e.g., fitness classes taught in converted teacher's lounge and nutrition/meal prep classes offered) • Offering classes in alternate locations
Space is limited in school buildings	Potentially limited opportunities to reach students/staff/community members	• Repurposing existing rooms (e.g., ABL lab, Neuronasium, and HS teachers' lounge) • Using floor space in the hallways (e.g., Sensory Pathway)
Lack of buy-in and competing priorities	Hinders program from expanding or being utilized fully	Developed buy-in from critical players • *New principal:* Worked with superintendent, shared the purpose and the supporting research • *New teachers:* Incorporated C2BF as part of standard orientation, including providing C2BF swag, and making it an invitation to “be part of the C2BF family”
Resistance to change	Difficulty implementing programming and initiatives, especially when required significant staff buy-in	• Phased in C2BF • Worked to earn trust early • Used a participatory/engaged approach to allow those who would implement to have a voice (e.g., cafeteria staff)
**COVID challenges**		
Child nutrition procedures no longer functioned during the at-home phase	Students who relied on school food faced increased food insecurity; which hindered C2BF cafeteria objectives	• Used POP Bucks for students to pick up lunches at Farmers Market • School lunch moved to delivery for weekday + weekend meals
Community offerings canceled during at-home phase	C2BF was unable to offer in-person classes and workshops	• Held a highly successful community walking challenge to benefit the Food Pantry • Offered online strength training programs in 12- and 8-week formats. • Moved much content online, which expanded reach
Teacher exhaustion and low morale	Teachers were less able to engage in healthy behaviors, model healthy behaviors for students, and support C2BF programming	• Show extra gratitude to teachers • Organized farmer's markets at the school with teacher certificates for free produce • RCPS provided extra compensation when teachers had remote learners in class
Student poor mental health	Students encountered additional trauma and stress due to COVID	• Created calming corners • Shifted disciplinary protocols to use calming corners before referral • Set up mindful minute push-ins

### 3.5. Keys to C2BF

The full evaluation results of various facets of C2BF is beyond the scope of this paper and will be described in detail elsewhere. However, we provide an overview here of the broad approaches we used to discover what has been happening in C2BF and why. We provide the results of a strategic planning activity in 2018 as a bridge to discuss the critical elements of C2BF identified by team members and stakeholders in 2022.

#### 3.5.1. Evaluation

External evaluators have partnered with RCPS and the funding organization to undergo process, developmental, implementation, and outcome evaluations of C2BF. Many individual interviews and focus groups have been held over the course of 4 years to gather data from C2BF team members, stakeholders, and participants. Power hierarchies were considered when focus groups were used, and individual conversations were held with each contributing participant to confirm interpretations (42). To allow triangulation [(43), p. 301], additional data were gathered from RCPS archival records (e.g., cafeteria records and behavioral referrals), C2BF documents (e.g., newsletters and lesson plans), direct observation, physical artifacts, systems mapping, and behavior logs, in addition to objective measures. C2BF approaches are also being compared to existing scientific literature. One example of this literature review is to explain how movements that cause arms/legs to cross the midline of the body (like in ABL foundations) are important for reaching developmental milestones for fine and gross motor skills (44) and coordination of the two brain hemispheres (45, 46). Cross-case analyses (47) are in process to contrast C2BF with other school based wellness initiatives. The variety of data sources, investigators, theories, and methods [(43), p. 301] allows us to use triangulation to enrich, refute, confirm, and explain (42) while also reducing bias.

Early C2BF evaluations were often developmental and progressive [Parlett and Hamilton, 1976, as cited in (48)], and included approaches such as (1) a curriculum analysis (see https://sph.uth.edu/research/centers/cchi/resources for details on the Curriculum Evaluation Tool) based on behavioral theory to optimize lesson plans to encourage healthy behaviors; (2) an Enhanced Force Field Analysis for strategic planning to identify driving forces and restraining forces (Redelfs et al., in submission). We also incorporated outcomes measured via (3) reflectance spectroscopy (VEGGIE METER^®^, Longevity Link, Inc., Salt Lake City, UT) to objectively track changes in fruit and vegetable intake in children and RCPS staff over time (e.g., adding salad bars). Recent evaluation efforts include (4) assessing improvements in developmental gaps via children's kinesthetic movement in ABL, and (5) testing how participation in the ABL lab may affect academic achievement. Such knowledge could provide a mechanism for kinesthetic learning to address learning losses in young children.

In 2018, the external evaluators conducted an Exploratory Force Field Analysis (E-FFA) with the C2BF team, the Superintendent, and the funding program officer. Leveraging strategic planning, participatory approaches, and Appreciative Inquiry, E-FFA enables participants to evaluate driving (enabling) and restraining (barrier) forces, and generates useful data to make strategic decisions (Redelfs et al., in submission).^1^ The underlying principle is that change, progress, and growth can only be realized by either strengthening the driving forces or mitigating the restraining forces to change. We concluded with a participatory summative content analysis (49, 50) to increase relevance, consider context, and reduce evaluator bias as stakeholders interpreted the data and prioritized next steps. Comments related to the superintendent and funders were verified with participants later in individual conversations to reduce the effect of potential power dynamics on responses.

The C2BF team identified buy-in as one of the most influential positive forces, specifically buy-in from the superintendent, principals, and the funding organization, and to a slightly lesser degree from students and teachers who were early adopters. Strategic plans that were prioritized included creating division-wide action plans for health (including development of an RCPS wellness policy), participating in the committee to revise the Rappahannock County Comprehensive Plan and increase alignment, and increasing visibility (earned media, presentations, social media, etc.) as a vehicle for improving buy-in. The C2BF team was intentional about integrating these and other strategic actions into the proposed tasks for upcoming annual plans.

#### 3.5.2. Critical elements

Several years later (fall 2022), team members and the principal investigator (superintendent) were interviewed by the principal external evaluator regarding their experiences with C2BF and their perceptions of challenges and successes. Interviews were done individually to reduce power dynamics and potential bias; this would avoid the superintendent being present. After briefly reviewing previous evaluation findings and recommendations, the evaluator asked each person questions such as “What have been the most important factors for C2BF to grow/be sustained?” and “What made C2BF successful?” along with negatively framed questions like “What have been the greatest barriers for C2BF?” The full description and analysis of these interviews are reported in the paper with evaluation results. Interviewees' responses were thematically analyzed; the most common elements around success (and lack of failure) were: (1) buy-in and engagement from the RCPS superintendent, board, and other administrators; (2) a committed and supportive funding agency; (3) alignment with critical priorities within the district and the broader field of education; (4) buy-in from staff, students, and community members; (5) taking programming to the people (geographically and in terms of stages of change); (6) continually seeking feedback and ideas from stakeholders to address perceived needs and build buy-in; and (7) the breadth and inclusivity of the C2BF programming that provides “something for everyone” (potentially decreasing resistance). As an example, C2BF team members, school staff, and community members involved in programming frequently refer to the positive culture of health and wellness developed at RCPS.

The evaluators compared these elements with the driving and restraining forces from the 2018 Enhanced Force Field Analysis. Two elements stood out in this comparison and also aligned well with our evaluation experience as critical to program success and longevity: leadership engagement and aligned efforts.

#### 3.5.3. Leadership engagement

Intentionally involving leadership has been a priority effort of C2BF. As the principal investigator, the superintendent has been highly involved in C2BF since the beginning. The superintendent's leadership style is inspiring and persuasive instead of authoritative, allowing her to gently persuade stakeholders instead of forcing compliance and slowly building buy-in. She has successfully engaged leaders at all levels (e.g., school board, central administration, and principals) as well as a majority of staff and teachers, so there are actors at every level who care and contribute. Building relationships with the school board, principals, and county commissioners has helped cultivate broader support and has minimized pushback. The superintendent also has carefully integrated C2BF into school-level policy, from inclusion in the district comprehensive plan to changes in disciplinary processes that incorporate best practices for self-regulation.

Of the community residents interviewed in 2018, the majority (*n* = 11 of 16) made statements about perceiving the RCPS superintendent as having a deep commitment to the community, informed at least in part by C2BF. Generally, they expected this commitment would translate into a greater long-term impact of C2BF on the schools and community than they would expect otherwise.

#### 3.5.4. Aligned efforts

At the district level, C2BF was created to align with the already existing features of the health and wellness systems. At a local level, C2BF is aligned with (and incorporated into) the RCPS 5-year comprehensive plan as a mechanism through which the district can support student wellness; this includes C2BF support of a new telehealth clinic at the elementary school (there were no medical providers in the county prior to this clinic). RCPS provided input in the Rappahannock County Comprehensive Plan revisions in an effort to better align school and county efforts around health. At the state and national level, C2BF aligns with the Whole Child approach. The C2BF team has contributed to discussions with the Virginia School District Association around integrating a holistic approach to child health within the Commonwealth and, at a national level, with the US Department of Education and other educational associations.

## 4. Sustainability of C2BF

C2BF is an initiative that seeks to build a “culture of wellness” within its schools and the community by implementing holistic strategies across ecological levels. Although more complex, school initiatives that take a holistic approach and incorporate multiple, mutually supportive components across various ecological levels are more effective and sustainable (51). In addition, an essential strength of C2BF is the leadership and enduring commitment from the RCPS superintendent. While buy-in and engagement from principals, teachers, school nurses, and child nutrition staff are vital, the success and longevity of a district-wide health initiative depend on buy-in from upper-level leaders (e.g., the superintendent's office and/or school board) (52). Having the superintendent as the change agent has allowed C2BF to be consistently included in district-level policies and practices which would be critical to shifting organizational culture.

Programs such as C2BF will only benefit their intended audiences if the activities and practices can be sustained over time (53). Evaluators, the C2BF team, and key stakeholders agree that C2BF includes activities addressing the eight domains in the Program Sustainability Assessment Tool [PSAT; (53, 54)].

- Environmental support: C2BF has internal (e.g., superintendent, school board, teachers, and staff) and external (e.g., regional foundation and county leadership) champions and support.
- Funding stability: C2BF has external funding from a regional foundation and has integrated and aligned C2BF activities with school division functions [e.g., the C2BF staff member responsible for increasing public support and awareness (partially grant funded) also fills the role of RCPS media/community liaison (RCPS funded)]. Were the grant funding to go away, adaptations would have to be made to fully cover staff salaries through division funds.
- Partnerships: Community leaders and stakeholders are involved with C2BF (e.g., conversations between RCPS/C2BF and county officials; partnership with the Rappahannock Farmer's Market for Power of Produce bags; involvement with Rapp at Home—a senior village—to provide balance and modified fitness classes for aging residents).
- Organizational capacity: C2BF is integrated into standard RCPS operations, including child school nutrition, classroom teaching, PE classes, and communications.
- Evaluation: Ongoing process, developmental, implementation, and outcomes evaluations have been used to inform future planning and strategic development.
- Adaptation: C2BF has adapted to changing priorities (e.g., shift from child obesity focus to holistic health) and environmental changes (e.g., adaptations in response to the COVID pandemic).
- Communications: C2BF includes a communications strategy for public awareness, engagement, and support. This strategy aligns with RCPS media relations, increasing sustainability through shared funding.
- Strategic planning: C2BF has applied evaluation results to address prioritized restraining forces (barriers), including ensuring roles and responsibilities are clearly defined and developing a sustainability plan.

Luke et al. (53) found that funding stability, communications, strategic planning, and sometimes political support were typically the domains with the lowest scores for the 592 community and government-sponsored programs they assessed. C2BF addresses all eight PSAT domains, including the less common domains, a positive indication of potential sustainability.

## 5. Limitations

The framework underlying C2BF presented here is the result of the application of many principles and behavioral theories within the specific context of RCPS and Rappahannock County. While the foundational principles themselves may be considered universal (e.g., the most effective approaches for changing behavior will address multiple levels of the ecological model) the specific application as seen in C2BF may not be completely generalizable to other school districts and communities. We sought to reduce bias by involving multiple evaluators, engaging in participatory analyses (55) to test alternative explanations, and triangulation.

## 6. Conclusion

C2BF is working to create a strong foundation for positive change in Rappahannock County. The C2BF program and other similar initiatives (51) provide examples and show the importance of holistic interventions that span multiple ecological levels. Multi-level interventions have been shown to have a better chance of positively impacting children's health and optimizing their learning ability. Future longitudinal research is needed to confirm the long-term outcomes of multi-level holistic programming on behavior, norms, culture, and health.

## Data availability statement

The original contributions presented in the study are included in the article/Supplementary material, further inquiries can be directed to the corresponding authors.

## Ethics statement

The studies involving human participants were reviewed and approved by the Institutional Review Board at the University of Texas El Paso or the Institutional Review Board at Brigham Young University, and by Rappahannock County Public Schools Administration. Written informed consent to participate in this study was provided by the participants' legal guardian/next of kin.

## Author contributions

SG and KD contributed to the conception of the C2BF initiative. SG, HJ, JT, and AB implemented the initiative and planned for upcoming phases. KD and ME provided strategic guidance. LW built the relationships that led to an evaluation partnership. AR organized and led the evaluations with assistance from DP. MS and AR wrote the first draft of the manuscript. JM wrote multiple sections of the manuscript and provided extensive revisions. All authors contributed to the manuscript revision, read, and approved the submitted version.

## Footnotes

^1^ https://scholarsarchive.byu.edu/facpub/5832/

## Supplementary material

Supplementary Video C2BF promotional video (copyright PATH Foundation 2019, used with permission).

Supplementary Material C2BF program delivery and reach.
