# Supplementary material for: The Commit to Be Fit framework: a community case study of a multi-level, holistic school-based wellness initiative in rural Virginia
Source: Front Public Health. 2023 Aug 16;11:1067454. doi: 10.3389/fpubh.2023.1067454 (PMC10468599; doi:10.3389/fpubh.2023.1067454)
Supplement: Supplementary Material — C2BF program delivery and reach. [file Table_1.pdf]

Supplementary file “Table 1: C2BF Program Delivery and Reach”

1 RCPS Enrollment and Employees

**RCPS Students**				
**Year**	**Elementary**	**High School**		**Total**
**2022-2023**	457	290		747
2021-2022	466	310		776
2020-2021	507	302		809
2019-2020	480	306		786
2018-2019	487	312		799
2017-2018	503	349		852
2016-2017	525	359		884
Average	**489**	**318**		**807**
**RCPS Employees**				
**Year**	**Elementary**	**High School**	**Other**	**Total**
**2022-2023***	77	53	34	164

* Note: staff numbers fluctuate by no more than 4 individuals per year

Redelfs, A. H., Smith, M., Merrill, J. A., Grimsley, S., Jenkins, H. E., Tederick, J. S., Butler, A. G., Dueck, K., Eastham Thomas, M., Perez, D. A., & Whigham, L. D. (2023). The Commit to Be Fit framework: A community case study of a multi-level, holistic school-based wellness initiative in rural Virginia. *Frontiers in Public Health*, *11*. https://doi.org/10.3389/fpubh.2023.1067454

2 C2BF Program activities, frequency and reach

Program activity	Responsible	Frequency of offering/use	Reach
**ABL**	ABL coordinator	Part of specials rotation (1 class every 6 days; 30 min)	Elementary (K-4)
**Daily 15**	Training by C2BF team; Teachers lead	Daily	PreK- 12 grades
**Calming corners**	Superintendent & principals, applied by teachers, support by C2BF teams	Constantly available	Primarily elementary (K-7)
**Mindfulness in classrooms**	Training by C2BF team; Teachers lead	Daily	PreK- 12
**Mental health innovators**	C2BF Coordinator	monthly meeting	Division-wide
**Kinesthetic learning: Push-ins**	C2BF team	Offered daily	PreK- 7
**Kinesthetic learning: Training & supporting teachers**	C2BF team	Annual since 2017	Division-wide
**Neuronasium**	C2BF team	Offered daily	High school (8-12)
**Nutrition/health elective**	Nutrition director; C2BF certified teacher	Each school year since 2017	High school (8-12)
**Discipline policy change**	Superintendent & principals, applied by teachers	daily, begun in 2021	Division-wide
**C2BF training during teacher onboarding**	Superintendent (set up), C2BF team lead	Annual since 2017	All new faculty
**Salad bars**	Nutrition director	HS daily (since ~2018), Elem 2/week (since ~2018)	Elem & HS: teachers, staff, students
**Taste testing**	Nutrition director; team	Monthly at ES, started monthly at HS in 2023	elementary students (PreK- 7); high school (8-12)
**Farm to school**	Nutrition director	Monthly	Division-wide
**Cooking club**	C2BF Coordinator; C2BF team	Annual	Elementary school (K-7)
**Water bottle refill stations**	Nutrition director	HS daily (installed 2018-2019)	HS (ES already had them)
**Farmer's Market at school**	C2BF Coordinator; C2BF team	Fall and spring	All staff (division wide)
**Power of produce (with farmer's market)**	C2BF Coordinator; C2BF team	April- October (weekly)	Division-wide
**Freezer meal prep classes**	Coordinator; team	Monthly	Staff and community
**Community health presentations**	Communications Specialist; C2BF team	Varies	Local, regional, state, national, and international
**Turkey trot**	Communications Specialist; C2BF team	Annual	All students, staff, community
**Kids fitness events**	C2BF team	Annual	K-12
**Community challenges**	Communications Specialist; C2BF team	Monthly	Staff and community
**Fitness classes (across all offerings)**	C2BF staff (certified instructors)	Weekly	Staff and community
**Yoga/mindfulness classes**	Contracted yoga teacher	Weekly	Staff and community
**Program newsletters**	C2BF communication specialist; team	Weekly	Subscribers, staff, shared on social media in community groups
**Health coaching**	C2BF certified health coaches	Offered weekly	Staff and community
**Educator hour**	C2BF Coordinator	Weekly during COVID	Staff and community
**Rapp at Home Balance classes**	C2BF staff (certified instructors)	Twice monthly (during COVID when C2BF classes not held)	Rapp at Home members

Redelfs, A. H., Smith, M., Merrill, J. A., Grimsley, S., Jenkins, H. E., Tederick, J. S., Butler, A. G., Dueck, K., Eastham Thomas, M., Perez, D. A., & Whigham, L. D. (2023). The Commit to Be Fit framework: A community case study of a multi-level, holistic school-based wellness initiative in rural Virginia. *Frontiers in Public Health*, *11*. https://doi.org/10.3389/fpubh.2023.1067454
